# Batch vs. continuous direct compression – a comparison of material processability and final tablet quality

**DOI:** 10.1016/j.ijpx.2023.100226

**Published:** 2023-12-21

**Authors:** B. Bekaert, P.H.M. Janssen, S. Fathollahi, D. Vanderroost, T. Roelofs, B.H.J. Dickhoff, C. Vervaet, V. Vanhoorne

**Affiliations:** a Laboratory of Pharmaceutical Technology, Department of Pharmaceutics, Ghent University, Ottergemsesteenweg 460, B-9000 Ghent, Belgium; b Department of Pharmaceutical Technology and Biopharmacy, University of Groningen, Antonius Deusinglaan 1, 9713 AV Groningen, the Netherlands; c DFE Pharma, Klever Strasse 187, 47568 Goch, Germany; d GEA Process Engineering, Keerbaan 70, B-2160 Wommelgem, Belgium

**Keywords:** Continuous manufacturing, Direct compression, Excipients, Raw material characterization, Material scinece, Multivariate analysis, Lactose

## Abstract

In this study, an in-depth comparison was made between batch and continuous direct compression using similar compression set-ups. The overall material processability and final tablet quality were compared and evaluated. Correlations between material properties, process parameters and final tablet properties were made via multivariate data analyses. In total, 10 low-dosed (1% *w*/w) and 10 high-dosed (40% w/w) formulations were processed, using a total of 10 different fillers/filler combinations. The trials indicated that the impact of filler type, drug load or process settings was similar for batch and continuous direct compression. The main differentiator between batch and continuous was the flow dynamics in the operating system, where properties related to flow, compressibility and permeability played a crucial role. The less consistent flow throughout a batch process resulted in a significantly higher variability within the tablet press (σ_CF_) and for the tablet quality responses (σ_Mass_, σ_TS_). However, the better controlled blending procedure prior to batch processing was reflected in a more consistent API concentration variability. Overall, the comparison showed the benefits of selecting appropriate excipients and process settings to achieve a specific outcome, keeping in mind some key differentiators between both processes.

## Graphical abstract

**Figure:**
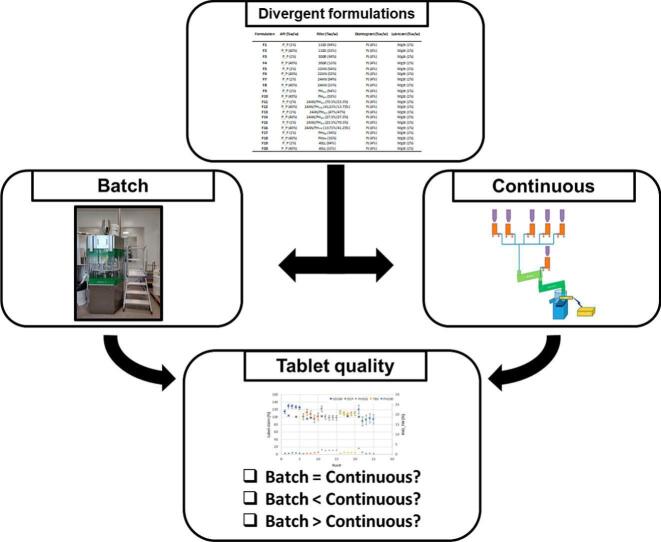

## 1 Introduction

The pharmaceutical industry is more and more looking at continuous manufacturing as the way forward. The well-documented advantages of continuous processing (i.e. cost efficiency; better product quality, e.g. uniform blend and tablet content uniformity; efficient production; ease of scalability) that accompany continuous processing are the main driver for this switch (Schaber et al., 2011; Lee et al., 2015; Tezyk et al., 2015; Ierapetritou et al., 2016; Nasr et al., 2017). Furthermore, regulatory agencies have put effort into the development of a new guideline (ICH, 2023) which specifically focuses on continuous manufacturing of drug substances and drug products. It has recently been adopted by the European Medicines Agency (EMA), coming in full effect by July 2023.

The ‘simplicity’ of the process (i.e. only 3 unit operations required) of (continuous) direct compression ((C)DC) makes it one of the most used production techniques. This is evident from the wide variety of studies published on this topic, ranging from fully integrated CDC systems (Järvinen et al., 2013a; Järvinen et al., 2013b; Simonaho et al., 2016; Van Snick et al., 2017a; Van Snick et al., 2017b; García-Muñoz, 2017 Roth et al., 2017; Van Snick, 2019; Galbraith et al., 2019; Galbraith et al., 2020; Bekaert et al., 2022c) to more in-depth investigations of each unit operation: feeding (Engisch and Muzzio, 2014; Blackshields and Crean, 2018; Hanson, 2018; Bostijn et al., 2019; Sacher et al., 2020; Bekaert et al., 2021; Bekaert et al., 2022b); blending (Pernenkil and Cooney, 2006; Portillo et al., 2008; Gao et al., 2011; Osorio and Muzzio, 2016; Palmer et al., 2020; Bekaert et al., 2022a; Jaspers et al., 2021); and tableting (Patel et al., 2006; Sun, 2010; Peeters et al., 2018; Van Snick et al., 2018a; Galbraith et al., 2020; Ervasti et al., 2020; Dhondt et al., 2022).

Even though these studies demonstrated the benefits of continuous direct compression, direct comparisons between batch-wise and continuous processes are limited. Research evaluating the blending unit operation in function of raw material properties (APIs as well as fillers), reported several limitations of batch-wise powder blenders including scaling difficulties, less flexibility, poorer content uniformity and higher dependency on the material property (Roth et al., 2017; Oka et al., 2017; Jaspers et al., 2021; Jaspers et al., 2022). When tablet quality (i.e. content uniformity, tensile strength and tablet weight) was compared after manufacturing on an integrated batch line versus a CDC line, differences between batch and continuous were observed for both high-dosed and low-dosed formulations (Karttunen et al., 2019).

Despite the research that has been performed, there are still gaps remaining in the comparison between batch and continuous direct compression. To the best of our knowledge, the scope of most research is either limited to one specific unit operation (potentially disregarding specific interactions that could occur in a fully integrated line) or when a fully integrated line was compared – the use of different equipment (e.g. different tablet presses between batch and continuous) could hamper the data interpretation.

In order to fill these gaps, current study processed both low (i.e. 1%) and high-dosed (i.e. 40%) formulations in a batch-wise manner and compared the observations to CDC data generated during a previous study (Janssen et al., 2023). The acquired data sets were produced using a similar setup (i.e. similar tablet press configurations), allowing for a more accurate comparison between batch and continuous direct compression compared to previous research studies. In the end, the overall processability and final tablet quality were evaluated and linked via multivariate data-analysis (i.e. partial least squares regression). Focus of the analyses was put on batch versus continuous and how these processes differ when divergent formulations were processed.

## 2 Materials

Table 1 gives an overview of the selected materials, including the supplier information and references to the abbreviations used in the paper. Paracetamol Powder was selected as a model API, having intermediate properties for the most important material characteristics (i.e. flow, cohesion, density, compressibility and particle size). This selection was based on the information available within the extensive material database developed by Van Snick et al. (2018b).

**Table 1:** Overview of selected materials, including the supplier information and abbreviations.

**Name**	**Material**	**Supplier**	**Code**
**SuperTab® 11SD**	Spray dried lactose	DFE Pharma	**11SD**
**SuperTab® 30GR**	Granulated lactose monohydrate	DFE Pharma	**30GR**
**SuperTab® 22AN**	Anhydrous lactose	DFE Pharma	**22AN**
**SuperTab® 24AN**	Granular anhydrous lactose	DFE Pharma	**24AN**
**SuperTab® 40LL**	Co-processed lactose-lactitol	DFE Pharma	**40LL**
**Pharmacel® sMCC90**	Silicified microcrystalline cellulose	DFE Pharma	**PHs**_**90**_
**Pharmacel® 102**	Microcrystalline cellulose	DFE Pharma	**PH**_**102**_
**Primojel®**	Sodium starch glycolate	DFE Pharma	**PJ**
**Magnesium Stearate**	Magnesium stearate	Sigma Aldrich	**MgSt**
**Paracetamol Powder**	Paracetamol powder	Mallinckrodt	**P_P**

## 3 Equipment

The study was performed on a stand-alone MODUL S tablet press (GEA, Halle, Belgium) where no PDV valve was present. In order to have a more profound comparison between the batch and continuous process, the rotary tablet press was configured in a similar fashion as the integrated MODUL S tablet press used in the study by Janssen et al. (2023) which collected the continuous process data.

The rotary tablet press was equipped with 38 punches with 8 mm round flat-face bevel-edge scored tooling (EURO B) and standard curved paddles in the forced feeder. As no blender was integrated during batch processing, the press was equipped with a feed chute connected to a stainless steel (10*L*) hopper with a rotating valve, connected to a level sensor. This part of the setup was the sole point of deviation from the MODUL S tablet press, integrated into the CDC-line, and was taken into consideration during the comparison of the generated data.

## 4 Methods

### 4.1 Blend preparation

In total, twenty blends were prepared: 10 low-dosed (1% *w*/w drug load) and 10 high-dosed (40% *w*/w) blends. Each blend consisted of paracetamol powder as the API, a filler or combination of fillers, Primojel® (4% w/w) as a super disintegrant and magnesium stearate (MgSt) as a lubricant (1% w/w). An overview of the blend compositions is given in Table 2.

**Table 2:** Overview of the processed blends.

**Formulation**	**API (%w/w)**	**Filler (%w/w)**	**Disintegrant (%w/w)**	**Lubricant (%w/w)**
**F1**	P_P (1%)	11SD (94%)	PJ (4%)	MgSt (1%)
**F2**	P_P (40%)	11SD (55%)	PJ (4%)	MgSt (1%)
**F3**	P_P (1%)	30GR (94%)	PJ (4%)	MgSt (1%)
**F4**	P_P (40%)	30GR (55%)	PJ (4%)	MgSt (1%)
**F5**	P_P (1%)	22AN (94%)	PJ (4%)	MgSt (1%)
**F6**	P_P (40%)	22AN (55%)	PJ (4%)	MgSt (1%)
**F7**	P_P (1%)	24AN (94%)	PJ (4%)	MgSt (1%)
**F8**	P_P (40%)	24AN (55%)	PJ (4%)	MgSt (1%)
**F9**	P_P (1%)	PH_102_ (94%)	PJ (4%)	MgSt (1%)
**F10**	P_P (40%)	PH_102_ (55%)	PJ (4%)	MgSt (1%)
**F11**	P_P (1%)	24AN/PH_102_ (70.5%/23.5%)	PJ (4%)	MgSt (1%)
**F12**	P_P (40%)	24AN/PH_102_ (41.25%/13.75%)	PJ (4%)	MgSt (1%)
**F13**	P_P (1%)	24AN/PH_102_ (47%/47%)	PJ (4%)	MgSt (1%)
**F14**	P_P (40%)	24AN/PH_102_ (27.5%/27.5%)	PJ (4%)	MgSt (1%)
**F15**	P_P (1%)	24AN/PH_102_ (23.5%/70.5%)	PJ (4%)	MgSt (1%)
**F16**	P_P (40%)	24AN/PH_102_ (13.75%/41.25%)	PJ (4%)	MgSt (1%)
**F17**	P_P (1%)	PHs_90_ (94%)	PJ (4%)	MgSt (1%)
**F18**	P_P (40%)	PHs_90_ (55%)	PJ (4%)	MgSt (1%)
**F19**	P_P (1%)	40LL (94%)	PJ (4%)	MgSt (1%)
**F20**	P_P (40%)	40LL (55%)	PJ (4%)	MgSt (1%)

Blends were prepared offline using a tumbling blender (Inversina, Bioengineering, Wald, Switzerland). The following protocol was followed: API, filler or filler combination and Primojel® were transferred to a 20 L stainless steel drum which was filled to 60% of its volume. This drum is rotated for 15 min at 25 rpm. Finally, MgSt is added, and mixed for 5 min at 15 rpm. In order to achieve the required amount of blend, multiple blends of the same formulation were prepared. Prior to tableting, the blends were pooled and stored in double low-density polyethylene bags that were kept sealed in a high-density polyethylene plastic container.

### 4.2 Blend characterization

2 kg of the prepared blend was used for characterization tests as described by Janssen et al. (2023). The data generated during these trials were used for data interpretation. Table 3 displays the descriptors, their abbreviation and applied characterization methods.

**Table 3:** Overview of blend descriptors, their respective abbreviation and applied characterization method.

**Characterization method**	**Descriptor**	**Abbreviation**
Flowpro	Flow through an orifice (= Flowrate) (mg/s)	FP
Helium pycnometry	True density	ρ_t_
FT4 powder rheometer	Compressibility (at 15 kPa) (%)	C_15kPa
	Permeability at 15 kPa (mbar)	k_15kPa
Laser diffraction	10% cumulative undersize of volumetric particle size distribution (PSD) (μm)	x_10_
	50% cumulative undersize of volumetric particle size distribution (PSD) (μm)	x_50_
	90% cumulative undersize of volumetric particle size distribution (PSD) (μm)	x_90_
Tapping device	Bulk and tapped density (g/mL)	ρb, ρt
	Hausner ratio (−)	HR
Ring shear tester	Effective angle of internal friction (°)	ϕint
	Cohesion (Pa)	τc
	Flow function coefficient (−)	ffc
	Major principal stress (Pa)	MPS
	Unconfined yield stress (Pa)	UYS
	Wall friction angle (°)	WFA
Compaction simulator	Compactability (MPa)	Comp. at plateau
	Yield strength (MPa)	PyS

### 4.3 Batch trials

#### 4.3.1 Experimental setup

To be able to compare batch and continuous processing, the tablet press settings were identical to the settings described in Janssen et al. (2023). A throughput rate of 20 kg/h was achieved by setting the turret speed at 50 rpm and aiming for tablets of 175.4 mg. The values of the paddle speed in the forced feeder were kept fixed throughout the experiments at 58 rpm and 70 rpm for paddle 1 and 2, respectively. A pre-compression force (PCF) of 1.5 kN with minimal displacement (PCD) (i.e. 0.2 mm) and a main compression force (MCF) of 10 kN were applied. The press was operated in a manual mode (identical to the continuous trials; Janssen et al., 2023) to avoid tablet rejection based on weight. Hence, the fill depth (FD) and compression roller heights (i.e. PCH and MCH) were adjusted to reach these setpoints.

Prior to start-up, the tablet press feed chute and stainless steel hopper were filled, followed by priming of the forced feeder. Priming was done by turning the paddles until the fill level in the feed chute was constant. During the start-up phase (± 15 min), tablet press settings (i.e. fill depth, pre-compression and main compression height) were adjusted in order to reach the required tablet weight and compression forces. When state of control was achieved (i.e. average tablet weight and compression forces are at setpoint), the tablet press was run for another five minutes after which samples were collected for 10 min via 30 consecutive grab samples during 20 s. Afterwards, the main compression force was changed to 5 and 15 kN. At each setpoint one grab sample (over a 20 s period) was collected after a run time of 5 min to ensure state of control.

#### 4.3.2 Data collection

Data were logged throughout the run and this information was combined with off-line generated data. An overview of the logged responses, off-line generated data and their abbreviations are given in Table 4.

**Table 4:** Overview of the collected responses and their respective abbreviation.

**Unit operation**	**Descriptor**	**Abbreviation**
Tableting	Fill depth (mm)	FD
	Pre-compression height (mm)	PCH
	Pre-compression displacement (mm)	PCD
	Pre-compression displacement variability (%)	σ_PCD_
	Main compression height (mm)	MCH
	Main compression height variability (%)	σ_CH_
	Main compression force variability (%)	σ_CF_
Tablet quality	Tablet tensile strength(MPa)	TS
	Tablet tensile strength variability (%)	σ_TS_
	Tablet weight variability (%)	σ_Mass_
	Tablet porosity (−)	ε_t
Off-line UV-VIS spectrophotometry	Label claim (%)	LC
	API concentration variability within a sample bag (%)	RSD_API%_within_
	API concentration variability between samples (%)	RSD_API%_between_

##### 4.3.2.1 Tablet press responses

Once the tablet press settings were optimized and a state of control was achieved, the values for fill depth, pre-compression and main compression height (PCH and MCH) were recorded. Additionally, the PCD (σ_PCD_) and MCF (σ_Force_) variability were collected via the MODUL S data-logging system. No ejection force values could be logged due to the unavailability of the logging system during the trial periods.

##### 4.3.2.2 Tablet analysis

The grab samples were used to determine the tablet weight (TW, mg), tablet crushing force (TCF; N), thickness (T; mm) and diameter (D; mm) of the tablets. Similar to the analysis performed by Janssen et al. (2023), 20 tablets from each unevenly numbered sample bag were randomly taken and analyzed using an automated tablet tester (Sotax AT50, Sotax, Basel, Switzerland). Based on these values, the tablet tensile strength (TTS; MPa) (**Eq.** 1), tablet weight variability (σ_Mass_) (**Eq.**2) and tablet porosity (ε_Tablet_) (**Eq.**3) was calculated:

(1) $$\mathrm{TTS}\;\left(\mathrm{MPa}\right)=\frac{2∙\mathrm{TCF}}{\pi ∙D∙T}$$

σ_Mass_ is the variability determined based on all tested tablets.

(2) $$\sigma_{\mathrm{Mass}}\;\left(\%\right)=\frac{\sqrt{\frac{\sum_{300}^{1}\left(\mathrm{TW}-\bar{\mathrm{TW}}\right)2}{300}}}{\bar{\mathrm{TW}}}\times 100$$

with $\bar{\mathrm{TW}}$ (mg) the average tablet weight.

(3) $$\varepsilon_{\mathrm{Tablet}}=1-\frac{\rho_{\mathrm{app}}}{\rho_{\mathrm{true}}}$$

with ρ_app_ the apparent density (i.e. tablet weight divided by its volume) and ρ_true_ the true density of the blend.

##### 4.3.2.3 API concentration variability

Off-line UV-VIS analysis was performed to determine the API content within a tablet. Three tablets, from sample bags with numbers 0; 5; 10; 15; 20; 25; and 30, were selected at random. Each tablet was homogenized in 50 mL distilled water, diluted 1/50 (for low-dosed blends) or 1/200 (for high-dosed blends) and measured at a wavelength of 243 nm using a UV spectrophotometer with a 1 cm cell (Shimadzu UV-1650PC, Shimadzu Corporation, Kyoto, Japan). The API concentration was determined via calibration curves which were developed through the analysis of five standards for each drug load (i.e. 0.50%; 0.75%; 1.00%; 1.25%; 1.5% for 1% drug load and 35.0%; 37.5%; 40.0%; 42.5%; 45.0% for 40% drug load). Based on the API concentration, the tablet label claim (LC) (**Eq.**4) and API concentration variability within and between sample bags (RSD_API%_within_, RSD_API%_between_) (**Eq.**5 and **Eq.**6, respectively) were calculated.

(4) $$\mathrm{LC}\left(\%\right)=\frac{\mathrm{Measured}\;\mathrm{API}\;\mathrm{concentration}\;\left(\%\right)}{\mathrm{Target}\;\mathrm{API}\;\mathrm{concentration}\;\left(\%\right)}x100$$

(5) $${\mathrm{RSD}}_{\mathrm{API}\%\_\mathrm{within}}\left(\%\right)=\frac{\sqrt{\frac{\sum_{3}^{1}\left(\mathrm{LC}-\bar{\mathrm{LC}}\right)2}{3}}}{\bar{\mathrm{LC}}}\times 100$$

(6) $${\mathrm{RSD}}_{\mathrm{API}\%\_\mathrm{between}}\left(\%\right)=\frac{\sqrt{\frac{\sum_{21}^{1}\left(\mathrm{LC}-\bar{\mathrm{LC}}\right)2}{21}}}{\bar{\mathrm{LC}}}\times 100$$

with $\bar{\mathrm{LC}}$ (%) the average label claim.

#### 4.3.3 Multivariate data-analysis

For the batch trials, all acquired blend properties (cf. Table 3; x-matrix), tablet press parameters (FD, PCH, PCD, σ_PCD_, MCH, σ_CH_, σ_CF_; x-matrix) and collected data (Tablet quality parameters and content uniformity responses; y-matrix) were used to develop PLS models via the SIMCA software (Version 16, Umetrics, Umeå, Sweden). One overall model, describing the batch trials for the 1% and 40% blends, was created and optimized in order to increase the goodness of fit (R^2^) and predictive ability (Q^2^). Additionally, the overall CDC-model developed in a previous paper (Janssen et al., 2023) was used to allow an in-depth comparison between both batch and continuous processing. Furthermore, an additional model containing data from both the batch and continuous trials was developed.

In order to have the best comparison, similar optimization steps as described by Janssen et al. (2023) were applied to the newly developed models. Both models were pre-treated prior to PLS regression via unit variance scaling and mean-centering. In the next step, log transformation was applied to non-normally distributed responses. Variables with a poor fit (i.e. R^2^Y < 0.3) and/or no significant correlation (i.e. coefficient plots contain 0) were removed from the models if their removal had a significant impact (i.e. R^2^Y increased with >0.1) on R^2^ and Q^2^. The tablet press parameter MCH was removed from the overall model (i.e. batch and continuous data combined), due to an observed mechanical issue in the continuous trials setup. This resulted in an offset in this value, making a comparison between the batch and continuous data impossible. However, the related main compression force could be applied and measured correctly, meaning that the observed issues did not impact the overall setup of the trials. Finally, an extra component was fitted to the model if: the component added new information; R^2^Y increased with >0.1; and/or Q^2^ increased.

## 5 Results and discussion

### 5.1 Batch trials – processability

**Fig. 1:**
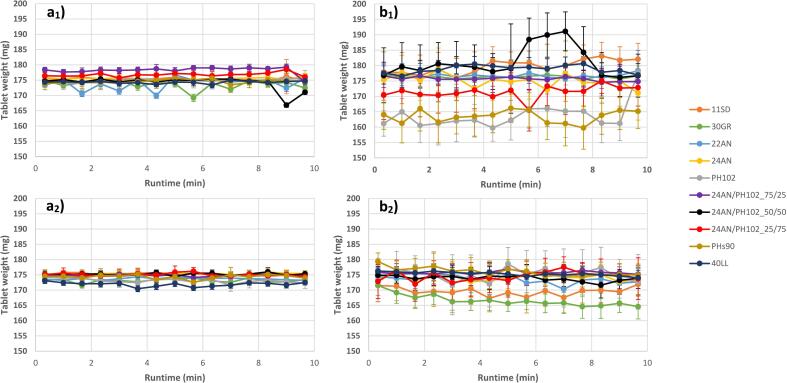
Average tablet weight (*n* = 20) in function of run time for the (1) batch and (2) continuous trials using blends with (a) 1% and (b) 40% drug load. Error bars depict 1 x standard deviation (SD).

Overall, the batch trials revealed that both low and high-dosed formulations were processable when aiming for a low (5 kN) and intermediate (10kN) main compression force. However, when aiming for higher compression forces (15 kN), it was observed that it was more difficult to maintain a constant applied force for the formulations with a high drug load (40%) as compression forces ranged between 12 and 28 kN, which originated from the higher die-filling variability for the poorer flowing blends (Fig. 1 **a**_**1**_; 1**b**_**1**_). Due to the safety settings of the tablet press (taking the breaking force of the tooling into consideration), main compression forces larger than 25 kN were not allowed. Processability issues could be resolved for example through the addition of a glidant, improving the overall product flow and die-filling consistency (e.g. optimizing the feed frame feeding configuration) (Soc et al., 1970; Sinka et al., 2004; Yaginuma et al., 2007; Parekh et al., 2023). However, this was not done during these trials, in order to allow a one-on-one comparison with the formulations processed during the continuous trials (Janssen et al., 2023).

**Fig. 2:**
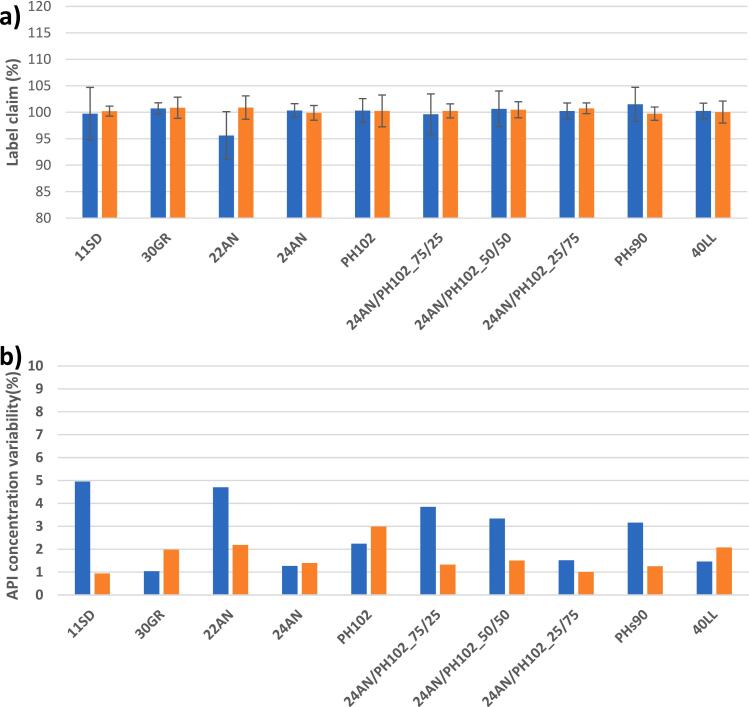
a) Average label claim and b) API concentration variability of batchwise produced tablets with a 1% (blue) and 40% (orange) drug load. (For interpretation of the references to colour in this figure legend, the reader is referred to the web version of this article.)

Another important aspect was the consistency in the drug load of the final tablet; i.e. tablet label claim and variability. The UV-VIS analysis data (Fig. 2) indicated that both the low and high-dosed formulations achieved their label claim with small variations (i.e. <5% RSD) with no visible trends within these variations (i.e. RSD_API%_between_). In contrast, the label claim variations during continuous processing varied up to 30% (Janssen et al., 2023). The low RSD values could be attributed to the batch process. Since the blends were manually produced off-line in a bin blender without the presence of loss-in-weight feeders introducing deviations (e.g. large deviations in the API feeder due to bridging; refill failures introducing variable feeder output), the risk of having large blend inhomogeneities was limited. Furthermore, the formulations were tableted the day after bin blending, reducing the risk of segregation. These observations could be further confirmed by the within sample API concentration variability (RSD_API%_within_), shown in **Supplementary section D (Table S1**). At specific timepoints within the continuous process (e.g. sample bags for 24AN – 1% and PH102–1%), large API concentration variations could be observed, which could be attributed to loss-in-weight feeder deviations.

### 5.2 Multivariate data-analysis

#### 5.2.1 Batch model

One PLS model with three principal components (PC) was generated with a goodness of fit (R^2^Y) and prediction (Q^2^) of 82.8% and 72.6%, respectively. The model regressed the blend properties and process settings (i.e. FD, MCH, PCH) against tablet press (i.e. σ_CH_, σ_CF_) and tablet quality (TS, σ_TS_, σ_Mass_, ε_t) responses. Label claim (LC) and API concentration variability (RSD_API%_within_ and RSD_API%_between_) data from UV-VIS analysis, as well as tablet press responses PCD and σ_PCD_ were excluded from the model due to low goodness of fit (i.e. < 0.3 R^2^Y). As mentioned above, the off-line produced blends resulted in limited to no variation (i.e. within the measurement error range) of the label claim (LC) and its variability (RSD_API%_within_ and RSD_API%_between_). Therefore, any deviation resulting from the blends could not be directly attributed to the blend properties or process settings,resulting in a low model fit. The responses PCD and σ_PCD_ were also not directly linked to specific blend properties or process settings since a PCD of 0.2 mm was targeted and was therefore operator dependent (i.e. operator chooses when the target value is reached). Hence, any observed variability is influenced by this unknown factor, resulting in a poor model fit.

**Fig. 3:**
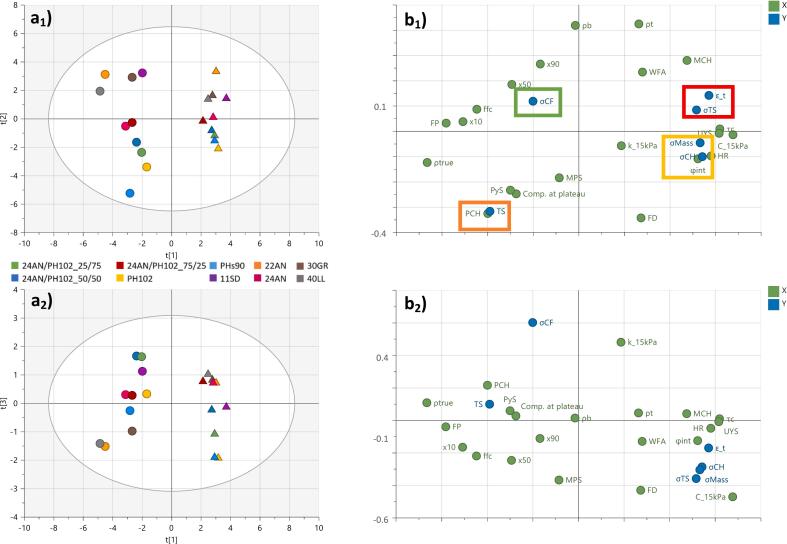
Scores (a) and loadings (b) plots for the batch model plotting: (1) PC1 vs. PC2; and (2) PC1 vs. PC3. •-labels and ▲-labels depict the 1% and 40% drug-loaded formulations, respectively. R^2^X_1_ = 0.462; R^2^X_2_ = 0.270; R^2^X_3_ = 0.065. Cluster 1 (green square); cluster 2 (red square); cluster 3 (yellow square); cluster 4 (orange square). (For interpretation of the references to colour in this figure legend, the reader is referred to the web version of this article.)

Correlations between the blend properties, process settings, tablet press and tablet quality responses were established through the scores and loadings plots (i.e. PC1 vs. PC2 and PC1 vs. PC3) depicted in Fig. 3.

##### 5.2.1.1 PC1 vs. PC2: formulation differentiation

Looking at the first two components (Fig. 3 **a**_**1**_ and 3**b**_**1**_), a clear horizontal split (i.e. split from left to right along the x-axis) can be observed based on the drug load (i.e. 1% on the left and 40% on the right). Formulations with a higher cohesivity (i.e. high τ_c_, HR and UYS; low ffc and FP) and compressibility (i.e. C_15kPa) were more located to the right side of the scores plot. This horizontal shift can be attributed to the higher cohesivity and compressibility of the API, which dominated the blend properties when a higher drug load is used. This dominating effect resulted in less filler differentiation within a cluster of blends with a higher drug load. Based on the horizontal separation, formulations containing more cohesive fillers such as microcrystalline cellulose (MCC) were located at the right side of the cluster, while the well flowing and lower compressible fillers (e.g. 40LL, 22AN) were at the left side (Van Snick et al., 2018b; Janssen et al., 2021).

The impact of the filler was mainly observed in the vertical direction of the scores plot, where a higher differentiation was possible for the 1% drug load, due to the lower impact of the API properties on the formulation. The main drivers along the vertical axis (i.e. PC2) are the properties related to density (ρ_b_ and ρ_t_) and powder compactability (PyS, Comp. at plateau).

Properties related to particle size distribution (x_10_, x_50_, x_90_) were the driver for a shift between the different formulations along both the horizontal (i.e. PC1) and vertical (i.e. PC2) axis, indicating that both the API and filler had a significant impact.

##### 5.2.1.2 PC1 vs. PC2: responses

Four clusters of responses can be observed in the loadings plot (Fig. 3 **b**_**1**_): cluster 1 describing the main compression force variability (σ_CF_) (green box); cluster 2 describing the tablet porosity (ε_T) and variability in tensile strength (σ_TS_) (red box); cluster 3 describing multiple tablet quality responses (σ_CH_, σ_Mass_) (yellow box); and cluster 4 describing the tablet tensile strength (TS) (orange box).

Cluster 1 is close to the origin and according to the coefficient plots does not have significant correlations along principal components 1 and 2 (see **Supplementary section A: Fig. S1 and S2**).

In cluster 2, the position of tablet porosity is based on its correlation with the true density. The API (ρ_True_ = 1.30 g/mL) has a lower true density than the fillers (ρ_True_ = 1.53–1.56 g/mL), resulting in a lower tablet porosity. Formulations that are located in the same direction as cluster 2 (i.e. top-right), are formulations containing a high API percentage and/or a filler with a lower true density (e.g. 11SD = 1.54 g/mL and 30 GR = 1.53 g/mL; Janssen et al., 2021). Furthermore, in the same cluster σ_TS_ can be found. This response is correlated to the cohesivity properties, indicating that a higher tensile strength variability is observed for formulations that have poorer flow properties due to the poorer die-filling of cohesive materials (Van Snick et al., 2018b). The higher variability in die-fill, combined with a fixed main compression roller height, will result in tablets with varying tensile strengths.

Cluster 3 contains the tablet quality responses that are correlated with the die-filling consistency. A higher variability of those responses (i.e. located on the right) is related to a lower die-filling consistency, due to highly cohesive formulations (i.e. located on the right of the scores plot) (Van Snick et al., 2018b).

Tensile strength as a tablet quality response is closely located to the properties linked to a high compactability. Formulations containing a plastically deforming material, such as MCC, form stronger compacts and are therefore more located in that direction, exhibiting higher tensile strengths (TS). The close proximity of TS to PCH can be related to the density of the formulations. The lower density fillers (at the opposite side of the density parameters on the scores plot) were also fillers that showed a high compactability. The higher fill depth which is the result of the lower density, required higher PCH values in order to achieve the target value. Therefore, PCH is located at the opposite side of density, close to the parameters linked to a high compactability.

Based on PC1 vs. PC2 it can be concluded that there is both a significant effect of API concentration and filler type on the outcome of the process (i.e. tablet press and tablet quality responses). At lower API concentrations, the filler type is dominant with MCC grades (e.g. PHs_90_, PH_102_) providing a higher compactability (i.e. higher TS), but also a higher variability of tablet quality responses due to its higher cohesivity (σ_CH_, σ_Mass_). On the other hand, if a lower variability is required, formulations containing a good flowing lactose grade (e.g. 22AN, 40LL) could be used. However, this will result in tablets with a lower tensile strength when produced at the same compression force. For formulations containing a high drug load, similar conclusions can be made. However, the impact of the filler will be less pronounced.

##### 5.2.1.3 PC1 vs. PC3

The location of σ_CF_ along the y-axis on the loadings plot (PC1 vs. PC3; Fig. 3 **b**_**2**_) and the component contribution plots (**Supplementary data B: Fig. S4c**) indicate that PC3 mainly describes correlations related to that response. Significant correlations can be found between σ_CF_ and material properties related to flow (ffc) and compressibility/permeability (C_15kPa/k_15kPa) (**Supplementary data A: Fig. S3**). The inverse correlation with flow can be attributed to the impact of flow on die-filling consistency, with a better die-filling consistency resulting in a more consistent compression force at a specific main compression height (lower σ_CF_). Furthermore, the easier volume reduction of formulations with a higher compressibility provides a buffer in order to obtain similar compression forces (lower σ_CF_) in case a variable powder volume is filled in the die. Permeability (k_15kPa) is anti-correlated with compressibility and therefore is located on the opposite side of the loadings plot closer to σ_CF_.

Overall, the observed correlations between σ_CF_ and blend properties show that a more consistent compression force (low σ_CF_) could be achieved using formulations exhibiting a highly consistent die-filling (i.e. well flowing and highly compressible materials).

#### 5.2.2 Comparison batch and continuous model

In general, the observations of the batch model were similar to the continuous model developed during the continuous trials (Janssen et al., 2023): a similar horizontal split based on drug load and vertical split based on filler type with only very limited shifts in the location for each formulation (see **Supplementary data C: Fig. S5**). Furthermore, correlations between the different responses, blend properties and process settings were similar with one main differentiator: σ_CF_. Although the same conclusions could be drawn for this response in both batch and continuous, an additional principal component in the batch model was required to describe the correlations with σ_CF_.

Overall, the observed similarities between the batch and continuous model show that although a different processing approach is used, similar interventions allow to improve the process performance. These interventions can be related to the selection of an optimal filler type, modifying the drug load and/or changing the process settings.

### 5.3 Batch vs. continuous

#### 5.3.1 Observations

Based on the batch (i.e. current study) and continuous (Janssen et al., 2023) trials, it was evident that the manufacturing mode affected the outcome of the process. These aspects translated into differences related to processability, required process settings (of the tablet press) and tablet quality.

As mentioned by Janssen et al. (2023), several processability issues were observed during continuous manufacturing which were linked to either flow (during blending) or compressibility (during material supply: vacuum transport and top-up system of the feeder) of the material. As preblends were used during batch processing, no processability issues could occur prior to tableting. However, once the preblend was introduced in the tablet press, several issues were identified. Similar to the continuous process, the high compressibility and low fluidization potential of MCC formulations (i.e. PH_102_, lactose/MCC mixtures) resulted in a reduced flow throughout the system. This was clearly visible in the feed chute, where throughout the trial runs bridge formation required manual intervention (i.e. tapping the feed chute to break up the bridge). Additionally, runs with the higher drug load formulations also had persistent issues with maintaining a consistent flow through the system (i.e. bridge/rathole formation). This could be attributed to a combination of poorly flowing materials and the powder pressure from the filled hopper. These phenomena translated during batch manufacturing into the inability to achieve a stable process when a main compression force of 15kN was required (cf. **5.1 Processability**).

**Fig. 4:**
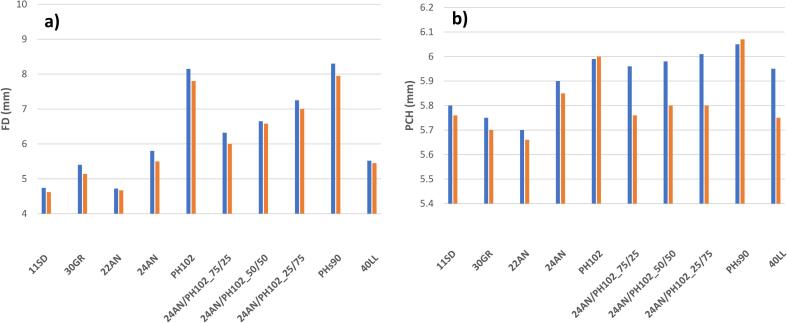
Overview of tablet press responses required to achieve the target tablet weight and pre-compression force for 1% drug load formulations: (a) fill depth and (b) pre-compression height. Blue = batch; orange = continuous. (For interpretation of the references to colour in this figure legend, the reader is referred to the web version of this article.)

For the required tablet press settings (i.e. FD and PCH) to achieve the desired tablet weight and compression force, there is a significant difference visible for both batch and continuous tableting (Fig. 4; data for 40% drug load is given in **Supplementary section E: Fig. S6**). In order to achieve similar tablet weights and applied compression forces during batch processing, higher values for each of the corresponding tablet press settings were required. The increase in FD can be related to the powder flow throughout the system. During continuous manufacturing, the powder is continuously in motion as powder is introduced into the tablet press at the same rate as tablets are exiting the tablet press. As a result, the powder is presented to the tablet press in a more consistent and fluidized manner, requiring a lower fill depth to achieve a specific tablet weight. In contrast, during batch processing there is no continuous flow of incoming and outgoing powder. This results in a stationary powder bed that can compact in the feed chute, forming bridges or ratholes, thus reducing the consistency of the blend density and consistency of powder supply to the tablet press dies (observed in the larger tablet weight variability; Fig. 1). In order to cope with this, a higher FD is required. The higher FD directly impacted the other tablet press settings, since a potentially larger powder volume prior to compression will require a larger PCH.

Both pure MCC formulations (i.e. containing PH_102_ and PHs_90_) differed from other formulations as a higher PCH was required during continuous processing. It is hypothesized that this phenomenon is related to the abovementioned flow issues and lower density of MCC within both the batch and continuous process. This translated in a large variability of the die-filling (i.e. large impact in terms of required FD) and a potential entrapment of air (i.e. rather limited impact in terms of FD) within the less dense powder bed. Since during continuous processing, the powder was more fluidized, there was a higher probability of air being entrapped within the powder bed during die filling, resulting in a pressure build-up during pre-compression. Therefore, the required pre-compression force (cf. **4.3.1 Experimental Setup**) was reached at higher PCH values. This phenomenon was not observed for the formulations combining MCC with lactose, which could indicate that lactose improved powder flow and density of the mixture (i.e. lactose fills the holes between the MCC particles).

As a final differentiator between batch and continuous, several tablet quality responses were compared, with significant differences for tablet weight variability (σ_Mass_) and tablet API concentration variability (RSD_API%_between_). In general, the tablet weight variability showed similar trends for both processes, with a higher variability observed for the higher drug loads (i.e. 40%) (Fig. 1 **b**_**1**_ and 1 **b**_**2**_). Furthermore, a higher variability in tablet weight was observed for similar formulations in batch trials compared to continuous trials (Fig. 1 and Table 5**)**. Similar to the other observations, these differences could be linked to powder flow dynamics within the process. Keeping in mind that we are working with an identical setup during batch and continuous processing, certain optimizations (e.g. optimization feed frame configuration and paddle wheel settings) could help in improving powder flow dynamics.

**Table 5:** Minimum and maximum tablet weight variability observed for both batch and continuous trials.

**σ**_**mass**_**– Batch**		
**Drug load**	**Min**	**Max**
**1% w/w**	0.60%	1.50%
**40% w/w**	1.80%	5.70%
**σ**_**mass**_**– Continuous**		
**1% w/w**	0.50%	1.20%
**40% w/w**	1.30%	4.30%

The label claim was impacted by the manufacturing mode. Table 6 shows that in general a higher deviation from the target label claim is observed for tablets produced in a continuous manner. This observation was attributed to the different methods used to prepare the blends: off-line bin blending process during batch processing (yielding RSD values between 0.9% and 5%) compared to the continuous in-line blending process during continuous direct compression. The latter blending process was also affected by the feeder performance where the overall feeding consistency has a large impact on the blend variability (Bekaert et al., 2022a; Jaspers et al., 2021; Jaspers et al., 2022). The problems in feeding consistency resulted in RSD values between 0.7% and 25%. Although blend uniformity deviated more during continuous manufacturing, both processes were capable of achieving tablets with the target label claim (i.e. 100% LC was reached, keeping the SD in consideration). However for formulations with high standard deviations (>5%), changes to the current (standard) feeder setup (e.g. different screws, operating at higher screw speeds, …) should be implemented to drastically reduce the variability and overall consistency in tablet label claim (Bekaert et al., 2021).

**Table 6:** Average label claim (%) + Standard Deviation (%) for all formulations during batch and continuous processing.

**Formulation**	**Description**	**LC – Batch (%) ± SD (%)**	**LC – Continuous (%) ± SD (%)**
**F1**	11SD_1%	99.8 ± 4.9	99.0 ± 5.8
**F2**	11SD_40%	100.2 ± 0.9	99.7 ± 1.8
**F3**	30GR_1%	100.7 ± 1.1	103.7 ± 2.5
**F4**	30GR_40%	100.9 ± 2.0	103.15 ± 3.6
**F5**	22AN_1%	96.9 ± 4.5	104.5 ± 3.5
**F6**	22AN_40%	100.9 ± 2.2	100.7 ± 1.5
**F7**	24AN_1%	100.4 ± 1.3	106.6 ± 10.9
**F8**	24AN_40%	99.9 ± 1.4	99.1 ± 2.9
**F9**	PH102_1%	100.3 ± 2.3	100.5 ± 19.6
**F10**	PH102_40%	100.3 ± 1.3	100.5 ± 1.3
**F11**	24AN/PH102_75/25_1%	99.6 ± 3.8	100.2 ± 1.6
**F12**	24AN/PH102_75/25_40%	100.3 ± 3.0	92.0 ± 5.5
**F13**	24AN/PH102_50/50_1%	100.7 ± 3.4	106.4 ± 12.8
**F14**	24AN/PH102_50/50_40%	100.5 ± 1.5	98.9 ± 1.5
**F15**	24AN/PH102_25/75_1%	100.2 ± 1.5	103.0 ± 2.8
**F16**	24AN/PH102_25/75_40%	100.8 ± 1.0	100.1 ± 3.9
**F17**	PHs90_1%	101.5 ± 7.2	100.8 ± 1.4
**F18**	PHs90_40%	99.7 ± 1.3	99.6 ± 7.1
**F19**	40LL_1%	100.3 ± 1.5	103.1 ± 2.0
**F20**	40LL_40%	100.1 ± 2.1	100.1 ± 2.5

#### 5.3.2 Multivariate data-analysis

Using the data gathered for the batch (cf. **5.2.2.1 Batch model**) and continuous model (Janssen et al., 2023), one overall PLS model was generated to confirm the conclusions made during the comparison. This model had three PCs with an R^2^Y and Q^2^ of 71.4% and 63.1%, respectively. The blend properties and relevant process settings (FD and PCH) were regressed against the overlapping responses (_CH_, σ_CF_, TS, σ_TS_, σ_Mass_, ε_t). As no feeders and continuous blenders were used during the batch processes and logging of the EF was not possible, the settings and responses related to those unit operations were removed. Furthermore, LC and RSD_API%_ data from UV-VIS analysis, as well as the tablet press responses PCD and σ_PCD_ were removed due to a poor goodness of fit (i.e. < 0.3 R^2^Y). As mentioned in **4.3.3 Multivariate data analysis**, the tablet press parameter MCH was removed from the overall model (i.e. batch and continuous data combined), due to an observed mechanical issue on the continuous trials setup.

**Fig. 5:**
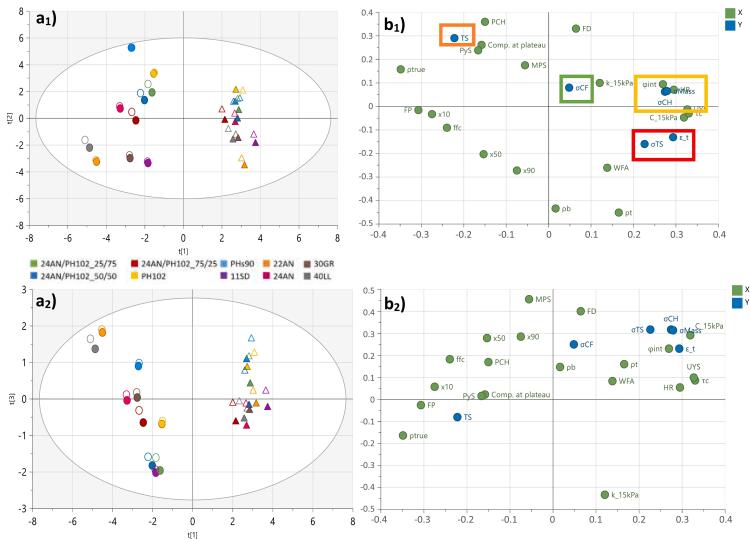
Scores (a) and loadings (b) plots for the batch and continuous model: (1) PC1 vs. PC2; and (2) PC1 vs. PC3. •-labels and ▲-labels depict the 1% and 40% drug-loaded formulations, respectively. Open labels = batch; closed labels = continuous. R^2^X_1_ = 0.46; R^2^X_2_ = 0.276; R^2^X_3_ = 0.0679. Cluster 1 (green square); cluster 2 (red square); cluster 3 (yellow square); cluster 4 (orange square). (For interpretation of the references to colour in this figure legend, the reader is referred to the web version of this article.)

Fig. 5 depicts the scores and loadings plots of the three components. The scores for the batch and continuous trials are clustered according to the formulation, indicating that similar correlations could be made for both processes. Small shifts in their location are related to the impact of the powder flow dynamics (i.e. along both PC1 and PC2). The loadings for PC1 vs. PC2 confirmed that the main drivers for both processes were the properties related to flow/cohesion (PC1: τc, HR, UYS, ffc and FP), density (PC2: ρb and ρt), particle size distribution (PC1 and 2: x_10_, x_50_, x_90_) and powder compactability (PC2: PyS, Comp at plateau). Principal component three depicted the correlation between the process responses and the blend permeability (k_15kPa), highlighting the abovementioned differences in powder flow dynamics. The scores plot (Fig. 5 **a**_**2**_) shows a clear vertical separation between the batch and continuous trials, indicating that – in general – blends processed in a batch manner had a lower fluidization potential (i.e. poorer powder flow dynamic), which was reflected in a less stable process (i.e. high compression force variability) and a poorer tablet quality (i.e. high tablet mass and tensile strength variations).

In general, the overall model defined on both batch and continuous process data confirmed the conclusions that were made during the individual trials and allowed to visualize the impact of both processes.

## 6 Conclusion

The use of a similar tablet press setup for a batch and continuous process allowed to perform an in-depth investigation of the similarities/differences between both processes. In general, both processes showed a lot of similarities in how the performance is impacted by a change in filler type, drug load or process setting. These changes were mainly affected by the flow dynamics in the operating system, with properties related to flowability, particle size, compressibility and permeability playing a crucial role. Furthermore, by varying the fillers, tablet quality responses such as σ_Mass_, σ_TS_ and TS could be tuned based on the required target. Due to the nature of batch processing (i.e. no continuous stream of powder), inconsistencies in the flow dynamics resulted in significantly larger variability within the tablet press (σ_CF_) and of the tablet quality responses (σ_Mass_, σ_TS_). However, a better overall tablet content uniformity was observed with batch processing thanks to the accurate pre-weighing of the different components before bin blending (in contrast to continuous processing, where there is an increased risk of fluctuations in composition when feeding of the individual components is not optimized; Janssen et al., 2023). This was reflected in a more consistent uniformity within the tablets. Although in practice, optimizing the feeder setup for each invidual formulation in the CDC-line could improve or overcome these issues.

Overall this comparison showed the benefits of selecting appropriate excipients and process settings in function of the drug load in formulations processed via batch as well as continuous direct compression.

## CRediT authorship contribution statement

**B. Bekaert:** Conceptualization, Data curation, Investigation, Methodology, Validation, Visualization, Writing – original draft. **P.H.M. Janssen:** Conceptualization, Formal analysis, Investigation, Methodology, Validation, Writing – review & editing. **S. Fathollahi:** Investigation, Methodology, Validation, Writing – review & editing. **D. Vanderroost:** Conceptualization, Resources, Writing – review & editing. **T. Roelofs:** Data curation, Validation, Writing – review & editing. **B.H.J. Dickhoff:** Conceptualization, Project administration, Resources, Supervision, Writing – review & editing. **C. Vervaet:** Project administration, Resources, Supervision, Writing – review & editing. **V. Vanhoorne:** Conceptualization, Project administration, Resources, Supervision, Writing – review & editing.

## Declaration of Competing Interest

The authors declare that they have no known competing financial interests or personal relationships that could have appeared to influence the work reported in this paper.

## Data Availability

Data will be made available on request.

## Appendix A Supplementary data

Supplementary material Image 1
