# Supplementary material for: Batch vs. continuous direct compression – a comparison of material processability and final tablet quality
Source: Int J Pharm X. 2023 Dec 21;7:100226. doi: 10.1016/j.ijpx.2023.100226 (PMC10792456; doi:10.1016/j.ijpx.2023.100226)

Supplementary data

Supplementary section A

Figure S1: Coefficient plot for σ_{CF} describing potential correlations along PC1.

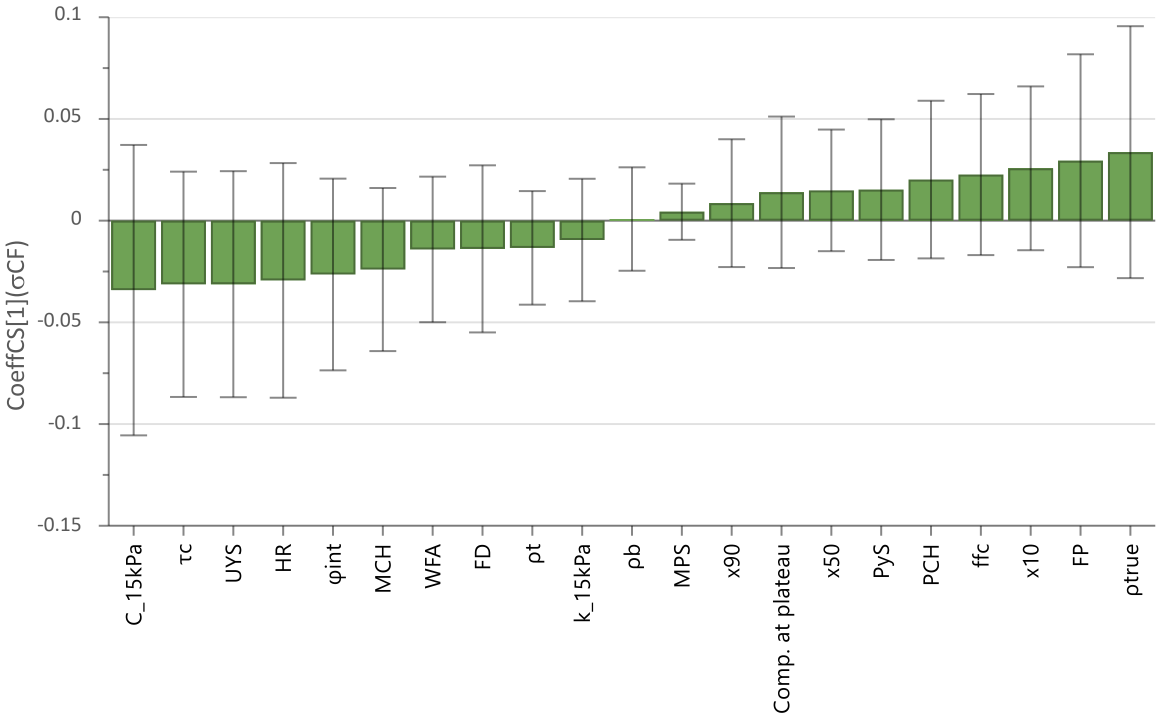

Figure S2: Coefficient plot for σ_{CF} describing potential correlations along PC2.

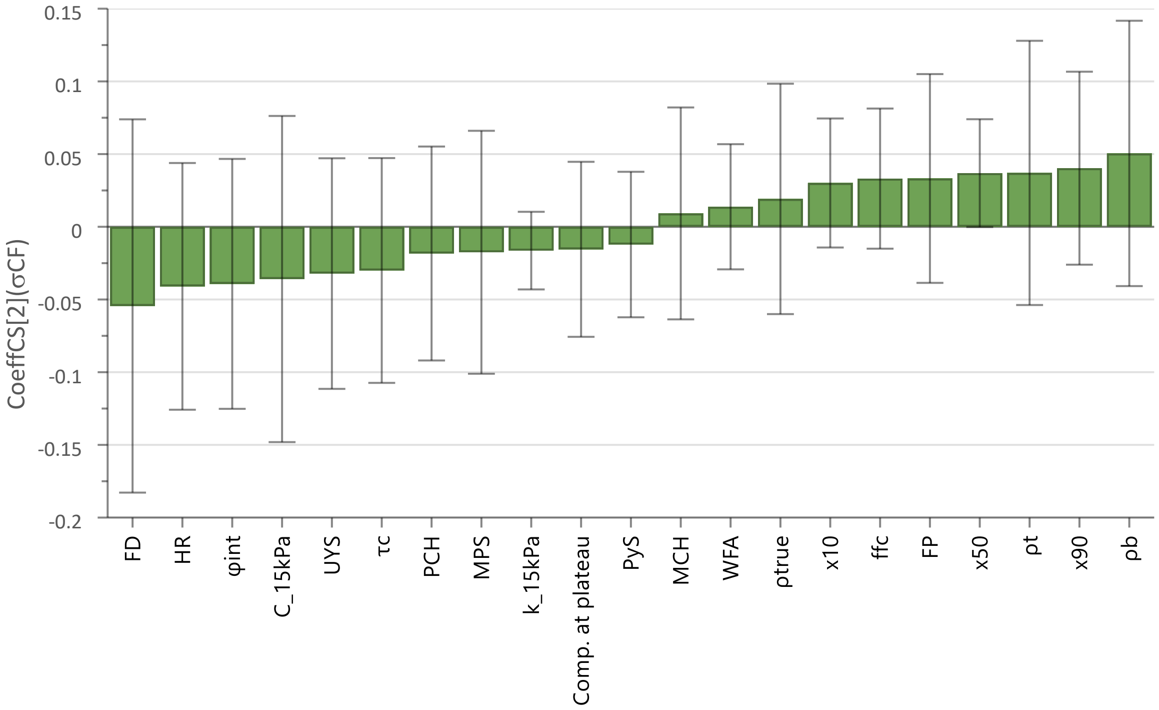

Figure S3: Coefficient plot for σ_{CF} describing potential correlations along PC3.

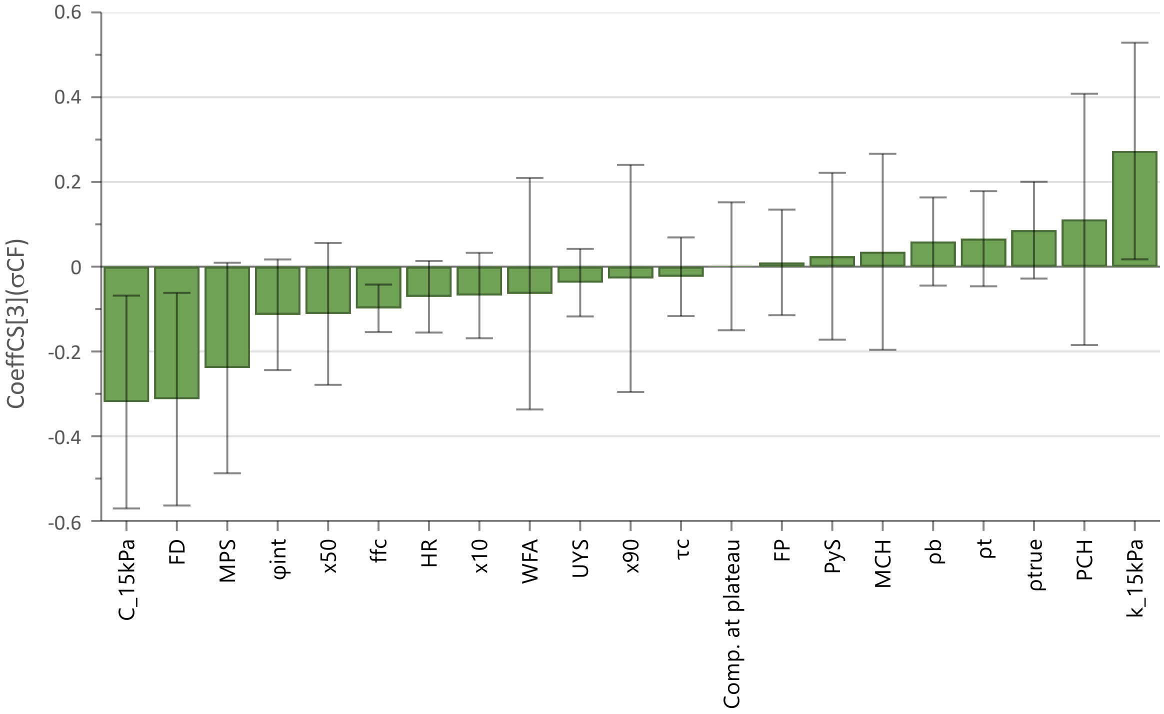

Supplementary section B

Figure S4: Component contribution plot for (a) PC1; (b) PC2; (c) PC3

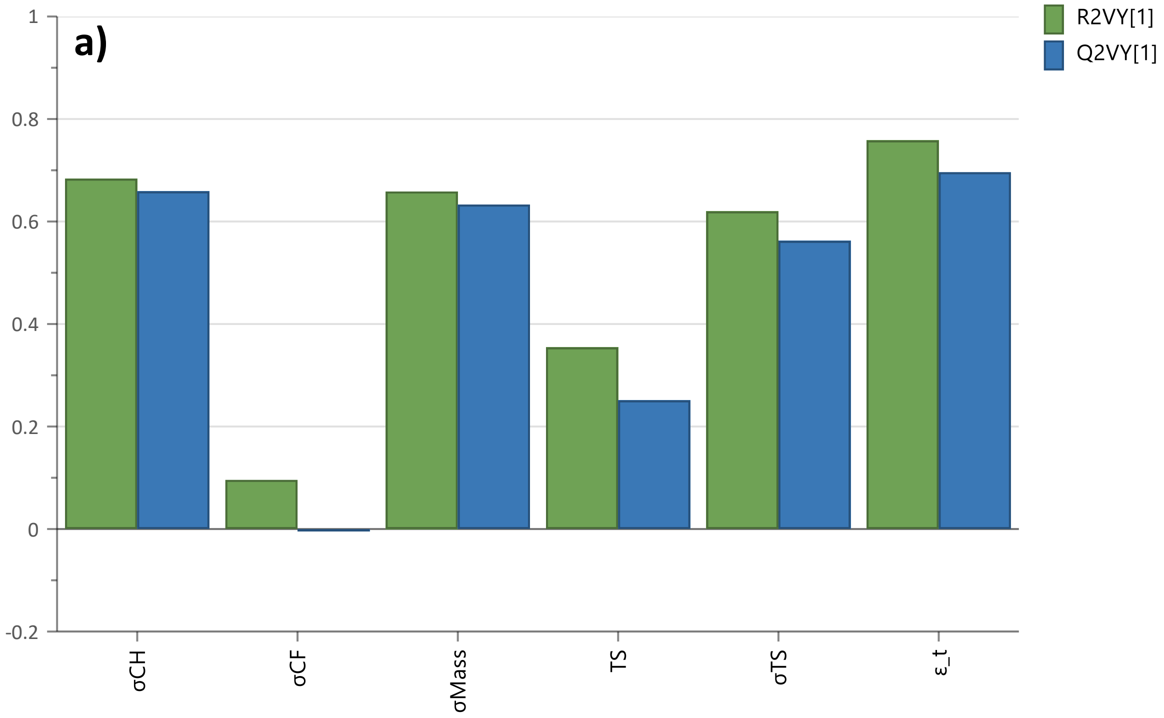

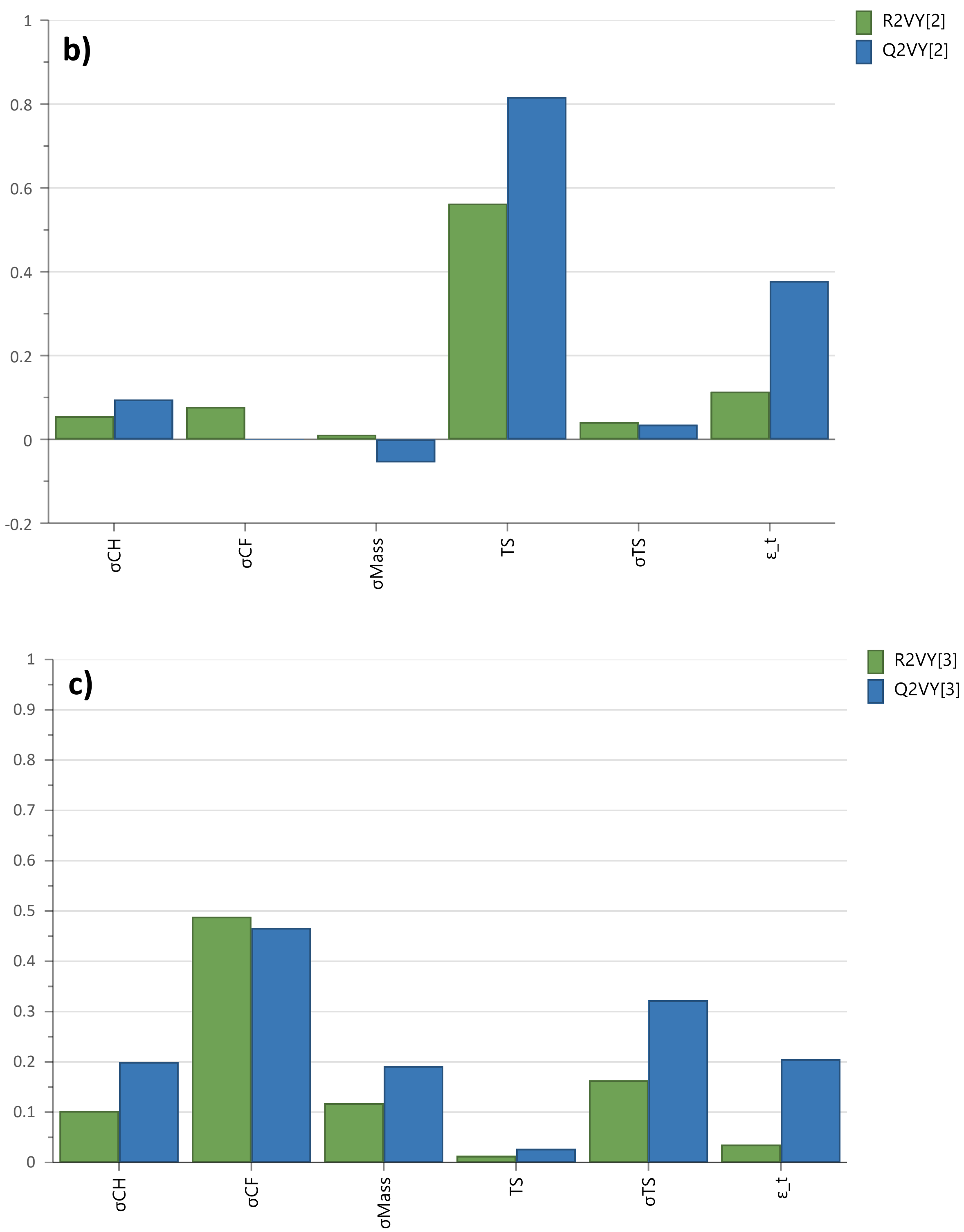
b)
R2VY[2]
Q2VY[2]
1
0.8
0.6
0.4
0.2
0
-0.2
σCH
σCF
σMass
TS
σTS
ε_t
c)
R2VY[3]
Q2VY[3]
1
0.9
0.8
0.7
0.6
0.5
0.4
0.3
0.2
0.1
0
σCH
σCF
σMass
TS
σTS
ε_t

Supplementary section C

Figure S5: Figure adapted from **Janssen et al. (2023)** describing the scores for the same twenty formulations evaluated on a continuous direct compression line. Shared with permission of the author(s).

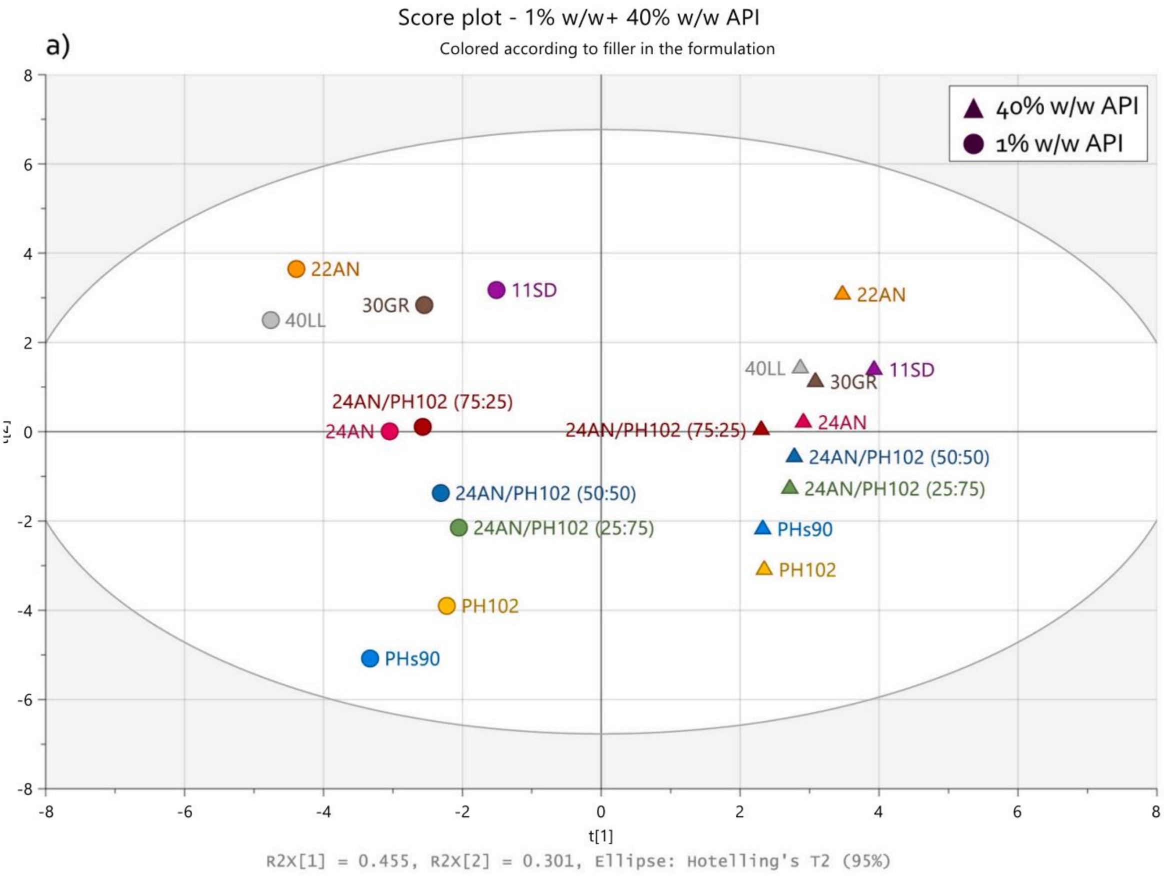

Supplementary section D

Table S1: Within variability values for the label claim for samples processed during both the batch and continuous trials.

Sample bag#	LC - Batch Within Variability (%)	LC - Continuous Within Variability (%)
	**11SD – 1%**	
**1**	2.30	3.68
**5**	6.25	3.15
**10**	1.25	3.60
**15**	1.60	8.51
**20**	2.56	10.55
**25**	2.18	0.71
**30**	2.99	5.97
	**11SD – 40%**	
**1**	0.73	1.91
**5**	1.68	0.61
**10**	1.27	0.19
**15**	1.03	1.38
**20**	0.85	3.14
**25**	0.46	1.04
**30**	1.42	0.32
	**30GR – 1%**	
**1**	0.51	2.70
**5**	0.78	1.55
**10**	0.50	2.86
**15**	0.96	0.80
**20**	0.84	1.14
**25**	0.68	2.70
**30**	0.87	4.47
	**30GR – 40%**	
**1**	0.37	1.01
**5**	1.43	2.92
**10**	0.60	2.90
**15**	3.84	2.36
**20**	1.73	1.01
**25**	1.83	2.00
**30**	5.22	1.67
	**22AN – 1%**	
**1**	1.23	8.40
**5**	3.25	3.43
**10**	4.88	4.51
**15**	5.34	1.77
**20**	5.93	2.12
**25**	2.16	1.45
**30**	5.78	1.80

Sample bag#	LC - Batch Within Variability (%)	LC - Continuous Within Variability (%)
	**22AN – 40%**	
**1**	2.61	0.42
**5**	3.03	0.57
**10**	2.07	0.72
**15**	1.68	2.37
**20**	1.40	2.15
**25**	1.42	0.09
**30**	2.08	1.45
	**24AN – 1%**	
**1**	1.40	23.68
**5**	0.21	9.66
**10**	0.30	11.24
**15**	0.56	28.75
**20**	0.69	5.20
**25**	0.94	1.09
**30**	1.85	8.40
	**24AN – 40%**	
**1**	1.71	2.44
**5**	0.69	5.62
**10**	1.19	2.74
**15**	0.88	2.08
**20**	1.53	1.87
**25**	1.14	2.16
**30**	0.39	2.58
	**PH102 – 1%**	
**1**	2.18	13.20
**5**	1.30	11.27
**10**	2.35	16.80
**15**	2.82	13.29
**20**	2.85	21.22
**25**	8.65	25.77
**30**	1.00	7.31
	**PH102 – 40%**	
**1**	1.53	1.12
**5**	1.53	0.41
**10**	8.52	1.22
**15**	1.21	0.64
**20**	1.33	0.86
**25**	1.51	3.07
**30**	1.99	1.30

Sample bag#	LC - Batch Within Variability (%)	LC - Continuous Within Variability (%)
**24AN/PH102 (75/25) – 1%**		
**1**	1.69	2.69
**5**	1.09	1.32
**10**	2.03	0.56
**15**	1.52	1.51
**20**	0.85	0.57
**25**	5.17	1.41
**30**	1.97	0.72
**24AN/PH102 (75/25) – 40%**		
**1**	0.70	7.50
**5**	1.88	3.58
**10**	0.98	4.11
**15**	0.49	4.77
**20**	1.05	4.43
**25**	0.60	2.27
**30**	1.72	3.54
**24AN/PH102 (50/50) – 1%**		
**1**	1.36	0.29
**5**	1.06	0.16
**10**	0.87	3.91
**15**	5.48	9.50
**20**	2.63	13.58
**25**	1.56	6.52
**30**	1.32	3.99
**24AN/PH102 (50/50) – 1%**		
**1**	0.41	0.86
**5**	0.95	2.15
**10**	1.69	2.22
**15**	1.65	0.85
**20**	1.44	1.47
**25**	2.33	1.50
**30**	3.11	1.11
**24AN/PH102 (25/75) – 1%**		
**1**	3.63	1.98
**5**	1.32	3.20
**10**	2.01	2.24
**15**	0.60	2.36
**20**	0.43	1.32
**25**	1.26	3.38
**30**	0.94	2.30

Sample bag#	LC - Batch Within Variability (%)	LC - Continuous Within Variability (%)
**24AN/PH102 (25/75) – 40%**		
**1**	1.11	1.58
**5**	0.46	3.26
**10**	1.15	5.21
**15**	0.70	3.82
**20**	0.82	2.38
**25**	1.50	4.03
**30**	0.33	3.52
**PHs90 – 1%**		
**1**	3.20	1.02
**5**	3.03	0.82
**10**	3.99	0.95
**15**	1.65	0.70
**20**	10.36	0.76
**25**	7.05	0.66
**30**	2.61	1.85
**PHs90 – 40%**		
**1**	0.75	1.32
**5**	0.30	2.80
**10**	1.96	8.06
**15**	2.12	3.24
**20**	1.76	1.15
**25**	1.13	1.15
**30**	0.47	7.01
**40LL – 1%**		
**1**	2.77	2.19
**5**	3.12	0.41
**10**	0.47	0.89
**15**	0.19	2.39
**20**	1.52	3.47
**25**	1.06	2.76
**30**	0.19	1.61
**40LL – 40%**		
**1**	1.43	0.83
**5**	1.25	1.97
**10**	1.33	2.40
**15**	0.71	1.14
**20**	3.23	1.01
**25**	0.53	0.67
**30**	2.99	1.34

Supplementary section E

Figure S6: : Overview of tablet press responses required to achieve the target tablet weight and pre-compression force for 40% drug load formulations: (a) fill depth and (b) pre-compression height. Blue = batch; orange = continuous.

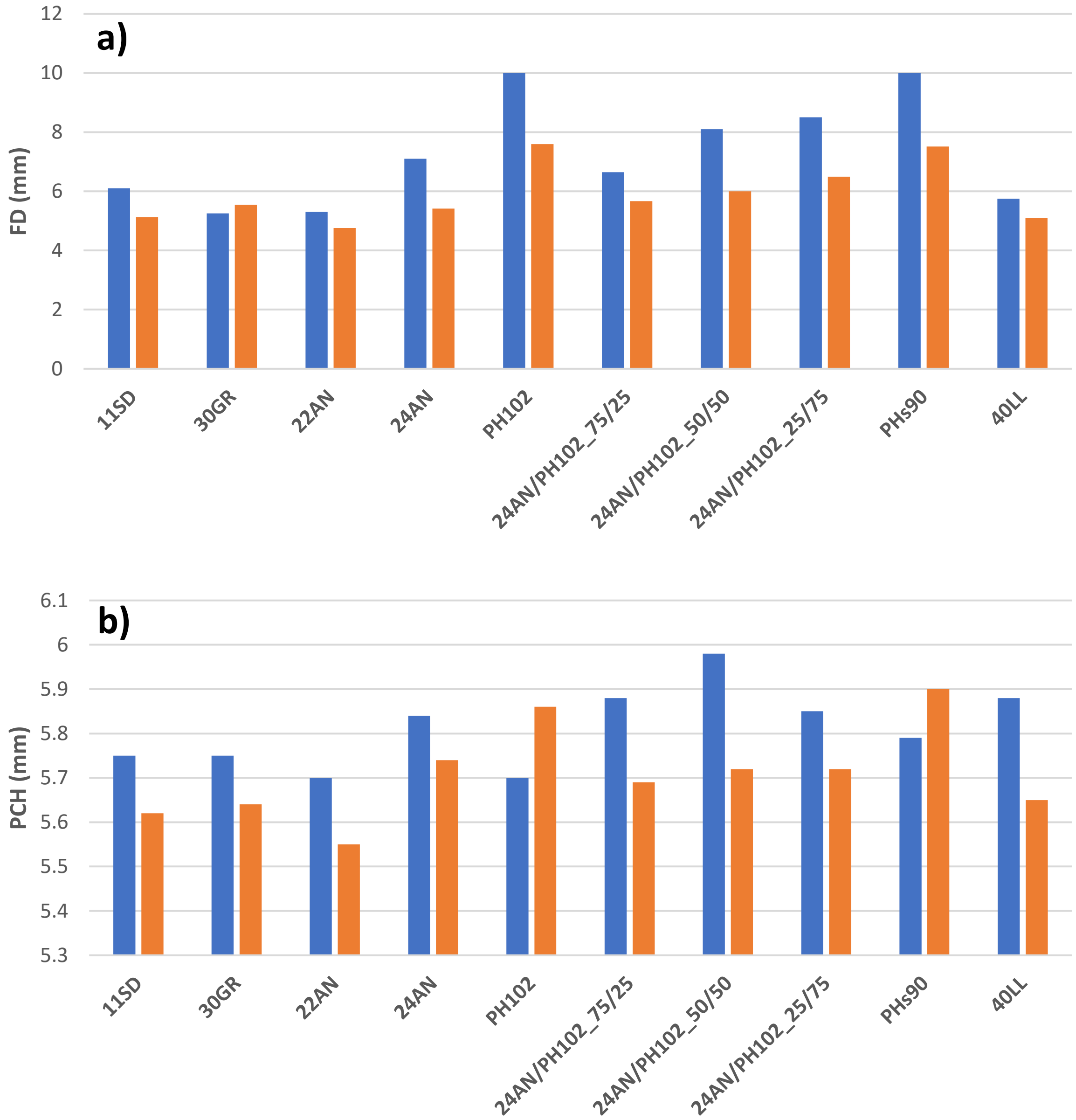

Supplement: Supplementary file 1 — Supplementary material [file mmc1.pdf]
